# Wearable- and Mobile App–Based Activity Pacing and Fatigue Management in Post–COVID-19 Condition: Exploratory Observational Study

**DOI:** 10.2196/91829

**Published:** 2026-05-29

**Authors:** Nana Yaw Aboagye, Mark R Baker, Kenneth Baker, Silvia Del Din

**Affiliations:** 1 NIHR Newcastle Biomedical Research Centre Translational and Clinical Research Institute Newcastle University Newcastle upon Tyne, England United Kingdom

**Keywords:** fatigue, digital biomarkers, post–COVID-19 condition, mobile health, activity pacing, wearables, long COVID

## Abstract

**Background:**

Post–COVID-19 fatigue affects millions worldwide; yet, evidence-based management strategies remain limited. Activity pacing, regulating activity to match available energy and minimize symptom exacerbation, may support symptom management, although optimal pacing approaches remain unclear.

**Objective:**

This study aimed to explore associations between activity pacing strategies delivered through a mobile app and daily fatigue levels in individuals with post–COVID-19 fatigue.

**Methods:**

In this exploratory observational study, 19 adults with post–COVID-19 fatigue used wearable devices (Fitbit Inspire 3) for objective activity monitoring and a mobile app (FatigueSense) to self-report daily symptoms (fatigue and energy levels) and optionally select activity pacing goals (light, balanced, or active) over a period of 3 months. We examined associations between pacing strategies and symptom outcomes by using mixed-effects linear models with random intercepts. Same-day outcomes (fatigue and energy reported on the day of goal selection) were analyzed controlling for age and sex. Next-day outcomes (fatigue and energy reported the day following goal selection) were analyzed controlling for age, sex, and prior-day symptoms.

**Results:**

Across 2182 observation days, participants self-selected pacing goals on 816 (37.4%) days, demonstrating symptom-responsive behavior with higher baseline fatigue on pacing days (mean score 1.74, SD 0.59 vs 1.58, SD 0.54 on nonpacing days on a 0-3 scale where 0=“none” and 3=“severe”; *P*=.004). On pacing days (584/816, 71.6% with complete data from 18 participants), active pacing was associated with reduced same-day fatigue (β=−0.34, 95% CI −0.49 to −0.19; *P*<.001) and increased same-day energy (β=8.8, 95% CI 4.7-12.9; *P*<.001) compared to light pacing. Balanced pacing also showed significant reductions in fatigue (β=−0.15; *P*=.008) and increases in energy (β=5.8; *P*<.001) compared to light pacing. Next-day effects were attenuated and nonsignificant (fatigue: β=−0.05, *P*=.53; energy: β=1.1, *P*=.61). Individual heterogeneity was substantial, with an intraclass correlation coefficient of 0.32, indicating that 32% of the variance was attributable to between-person differences. Among participants trying multiple strategies, 58.3% (7/12) showed meaningful responses (≥0.3-point fatigue reduction) to structured pacing strategies.

**Conclusions:**

Structured activity pacing strategies (active and balanced) were associated with improved same-day symptom management in individuals with post–COVID-19 fatigue. However, substantial confounding by indication (self-selection of pacing based on symptom state) and individual heterogeneity limit causal interpretation. These exploratory findings warrant testing in randomized controlled trials to establish efficacy and identify responder characteristics.

## Introduction

Postacute sequelae of SARS-CoV-2 infection (post–COVID-19 condition) affect an estimated 10% to 45% of individuals following acute COVID-19, with fatigue among the most prevalent and disabling symptoms [1-3]. Unlike postviral fatigue, which typically resolves within months, post–COVID-19 fatigue can persist for years, substantially impairing quality of life and functional capacity [4,5]. In this context, functional capacity refers to what individuals are physically capable of doing (eg, maximum walking distance in controlled conditions), whereas performance refers to what they actually do in daily life, which is often substantially lower in individuals with post–COVID-19 fatigue [3]. Despite the scale of this public health challenge, evidence-based management strategies remain limited [6].

Activity pacing, the strategic regulation of activity levels to align with available energy and limit symptom exacerbation, has been proposed as a self-management approach for postviral fatigue conditions [7,8]. Pacing can include a range of strategies, from reactive symptom-contingent approaches such as resting when fatigued or avoiding planned activities to proactive preplanned approaches, which involve setting activity goals in advance regardless of momentary symptoms [7,9,10]. While reactive symptom-contingent pacing may produce variable functional outcomes, preplanned, goal-directed pacing has been suggested to support symptom management by helping maintain physical capacity without triggering overexertion [9,10]. Structured pacing approaches may help improve activity tolerance and manage fatigue in individuals with chronic postviral fatigue, although the evidence remains limited and heterogeneous [7,9,10].

Post–COVID-19 fatigue may differ from myalgic encephalomyelitis/chronic fatigue syndrome (ME/CFS) in certain characteristics, including fluctuating symptom trajectories and the potential for recovery in some individuals [11-13]. The participants in this study self-identified as individuals with fatigue as a result of long COVID and were not necessarily individuals with ME/CFS. Whether activity pacing supports symptom management in this population and which pacing strategies may be most effective remains unclear.

Digital health technologies that combine wearable activity monitors with symptom tracking apps provide opportunities to study activity and symptom patterns in real-world settings [14,15]. Wearable devices allow for the objective collection of physical activity metrics, including step count, active minutes, and heart rate, which can complement self-reported data and reduce reliance on recall [16]. Mobile symptom tracking apps enable real-time recording of symptom severity and fluctuations, providing detailed longitudinal data. Together, these technologies facilitate within-person analysis of the relationships between activity patterns and symptom outcomes in naturalistic settings.

We conducted an exploratory observational study to examine associations between activity pacing strategies and symptom outcomes in individuals with post–COVID-19 fatigue using the FatigueSense mobile app integrated with Fitbit (Google) wearable devices. We hypothesized that structured pacing approaches would be associated with improved symptom management compared to minimal or reactive pacing.

FatigueSense [17] is a free research app developed for symptom tracking and activity management in post–COVID-19 fatigue.

## Methods

### Study Design and Setting

This was an exploratory observational study examining real-world use of activity pacing strategies among adults with post–COVID-19 fatigue. The study was conducted between May 2024 and December 2024 at Newcastle University, Newcastle upon Tyne, United Kingdom. Participants were community-dwelling adults who engaged remotely with the study app from their own homes and daily environments; no in-person study visits were required after the onboarding process, and the study included a minimum intended follow-up duration of 3 months for each participant.

### Participants and Recruitment

Recruitment was conducted through 2 complementary channels. First, participants were recruited from an existing cohort of people with post–COVID-19 fatigue previously engaged in patient and public involvement and engagement activities at Newcastle University. Patient and public involvement and engagement participants had previously been involved in shaping the research agenda around post–COVID-19 fatigue, and those who expressed interest in further participation were approached by the research team via email with a participant information sheet. Second, recruitment was extended through online patient communities and social media platforms (including long COVID–specific forums and advocacy groups), where study advertisements were posted with a link to the participation information and contact details.

Eligibility criteria were (1) self-reported post–COVID-19 fatigue persisting for 12 weeks or more after acute COVID-19, (2) age of 18 years or above, and (3) access to a smartphone. Participants provided informed consent to trial the app.

### Ethical Considerations

This study was approved by the Newcastle University Faculty of Medical Sciences Research Ethics Committee (protocol 47832-2023). All participants provided written informed consent prior to enrollment. Data were collected and processed in accordance with the General Data Protection Regulation and applicable institutional policies. Interested individuals who responded were provided with full study information and given time to consider participation before providing written informed consent electronically. No financial compensation was provided for participation.

### Data Collection

#### Objective Activity Monitoring

Participants wore Fitbit Inspire 3 devices continuously to capture objective physical activity metrics synchronized automatically with the FatigueSense app. We extracted daily step count and active minutes (minutes of moderate to vigorous physical activity) from Fitbit’s proprietary algorithms [16].

#### Symptom Reporting and Pacing Goal Selection

Participants used the FatigueSense mobile app (iOS and Android), developed as a research tool for post–COVID-19 symptom management research, to report daily symptoms and independently select activity pacing goals based on their own judgment of their energy levels and planned activities. The app did not provide algorithmic recommendations or prescriptive guidance; all pacing goal selections reflected participant choice. Participants received a notification prompt each morning (9 AM) to complete daily assessments, with reminders sent if not completed by noon. Assessments could be completed at any time until 11:59 PM on the same day and included the following: fatigue, measured on a 4-point ordinal scale (0=“none,” 1=“mild,” 2=“moderate,” and 3=“severe”) reported each day; energy, measured on a visual analog scale from 0 to 100 in 25-point increments (0=“no energy”; 100=“full energy”); pacing goal, comprising an optional morning selection of 1 of 3 predefined activity pacing levels (light, focusing on rest and minimal activity with a suggested target of <3000 steps; balanced, comprising moderate activity with regular breaks with a suggested target of 3000-7000 steps; and active, comprising a higher level of activity, including planned exercise, with a suggested target of >7000 steps); and goal achievement, measured using a “yes” or “no” indicator of whether the selected goal was met, assessed in the evening.

Participants could choose whether to set a pacing goal on any given day. Goal selection was not required, allowing for examination of pacing vs nonpacing behavior patterns.

### Outcomes

The primary outcomes were (1) same-day fatigue, fatigue rating on the day of pacing goal selection, and (2) same-day energy, energy rating on the day of pacing goal selection.

Secondary outcomes were (1) next-day fatigue, fatigue rating the day following pacing goal selection, and (2) next-day energy, energy rating the day following pacing goal selection.

### Data Analysis

#### Descriptive Statistics

We summarized participant characteristics, pacing goal selection patterns, and symptom distributions using appropriate descriptive statistics. For normally distributed continuous variables (assessed using Shapiro-Wilk tests), we reported means and SDs. For nonnormally distributed variables, we reported medians and IQRs. Categorical variables were summarized using frequencies and percentages.

#### Mixed-Effects Linear Models

To examine associations between pacing goal selection and symptom outcomes while accounting for the hierarchical structure of repeated measures nested within participants, we used mixed-effects linear regression with random intercepts for participants [18,19]. This approach was necessary because multiple observations from the same individual are not independent, violating assumptions of ordinary least squares regression. Mixed-effects models partition variance into between-person and within-person components, allowing us to estimate how pacing strategies are associated with symptoms while controlling for individual differences in baseline symptom severity.

We emphasize that our models examined associations rather than predictions; we sought to understand whether pacing strategies were associated with symptom outcomes after controlling for potential confounders, not to build predictive algorithms for future symptom states.

Model specification is as follows:

- Predictors of interest: pacing goal (active and balanced, with light as reference)
- Covariates: age group (35-44, 45-54, and 55-64 years) and sex (female and male) only for same-day outcomes and age and sex plus prior-day (same-day) fatigue and energy to control for autocorrelation for next-day outcomes
- Random effects: participant-specific intercepts

We did not control for contemporaneous symptoms in same-day models (eg, same-day energy when predicting same-day fatigue) as these represent simultaneous outcomes rather than potential confounders. Daily step count was not included as it may represent a mediator rather than a confounder. We report regression coefficients (β) with 95% CIs and *P* values.

We calculated the intraclass correlation coefficient to quantify the proportion of variance attributable to between-person vs within-person differences [20].

#### Confounding-by-Indication Assessment

To examine whether participants self-selected pacing goals based on symptom state, we compared baseline fatigue levels on days when pacing goals were vs were not selected using Mann-Whitney *U* tests.

#### Responder Analysis

We defined “responders” as individuals showing a 0.3-point or higher reduction in mean fatigue when using structured pacing (balanced or active) compared to their own light pacing days. This threshold may represent a meaningful change on the 4-point fatigue scale. Only participants who tried multiple pacing strategies could be included in within-person comparisons.

#### Data Inclusion Criteria

To ensure reliable within-person estimates, we included only participants who contributed 3 days or more of observation data with complete symptom reports and covariate information. One participant contributed only 1 day of data and was excluded from mixed-effects analyses, leaving 18 participants for complete-case analyses.

#### Data Analyses

All analyses were conducted in Python (version 3.13; Python Software Foundation) using *statsmodels* (version 0.14) and SciPy (version 1.11). Statistical significance was defined as a *P* value of less than .05.

### Reporting Guideline

This observational study was conducted and reported in accordance with the STROBE (Strengthening the Reporting of Observational Studies in Epidemiology) statement for observational studies. A completed STROBE checklist is provided as Multimedia Appendix 1.

## Results

### Participants

A total of 19 participants enrolled in the study and contributed 2182 observation days (mean 115; range 1-146 days per person). Of these 19 participants, 1 (5.3%) who contributed only 1 day of data was excluded from mixed-effects analyses due to insufficient data for reliable within-person estimates, leaving 18 (94.7%) for complete-case analyses (2181 observation days). The sample was predominantly female (16/19, 84.2%), with the following age distribution: 31.6% (6/19) were aged 35 to 44 years, 21.1% (4/19) were aged 45 to 54 years, 42.1% (8/19) were aged 55 to 64 years, and 5.3% (1/19) were aged 65 years or older. Baseline characteristics are presented in Table 1.

**Table 1:** Baseline characteristics of adults with post–COVID-19 fatigue enrolled in an exploratory observational study of wearable-supported activity pacing (Newcastle, United Kingdom; May 2024-December 2024; N=19).

Characteristic			Values
**Demographics, n (%)**			
	**Age (y)**		
		35-44	6 (31.6)
		45-54	4 (21.1)
		55-64	8 (42.1)
		≥65	1 (5.3)
	Sex (female)		16 (84.2)
**Observation period**			
	Days per participant, mean (range)		115 (1-146)
	Total observation days, n		2182
**Pacing goal setting, n (%)**			
	Days with pacing goal (n=2182)		816 (37.4)
	Days without pacing goal (n=2182)		1366 (62.6)
	**Among pacing days, days in which each type of pacing was selected (n=816)**		
		Light	255 (31.2)
		Balanced	414 (50.7)
		Active	147 (18)
**Strategy variety, n (%)**			
	Tried all 3 strategies		8 (42.1)
	Tried 2 strategies		8 (42.1)
	Tried 1 strategy		3 (15.8)
**Baseline symptoms (on pacing days), mean (SD)**			
	Fatigue (0-3)^a^		1.74 (0.59)
	Energy (0-100)^b^		49.1 (19.4)
**Fitbit activity metrics, mean (SD)**			
	Daily steps		6821 (5204)
	Active minutes		241 (98)

^a^Fatigue scale: 0=“none,” 1=“mild,” 2=“moderate,” and 3=“severe.”

^b^Energy scale: 0 to 100 in 25-point increments.

### Pacing Goal Selection Patterns

Participants selected pacing goals on 37.4% (816/2182) of the observation days and did not select goals on 62.6% (1366/2182) of the days. Among days with pacing goals, participants chose light pacing on 31.2% (255/816) of the days, balanced pacing on 50.7% (414/816) of days, and active pacing on 18% (147/816) of the days. After excluding observations with missing covariate data, of the 816 pacing days, 584 (71.6%) from 18 participants were available for complete-case mixed-effects analyses.

Pacing strategy use varied substantially across individuals. A total of 42.1% (8/19) of the participants tried all 3 strategies, 42.1% (8/19) tried 2 strategies, and 15.8% (3/19) used only 1 strategy. This variation reflects real-world behavioral heterogeneity in strategy adoption.

### Pacing Goal Selection and Baseline Symptoms: Evidence of Confounding by Indication

Participants demonstrated symptom-responsive behavior in their pacing goal selection. They were significantly more likely to select pacing goals overall on days with higher baseline fatigue: the mean same-day fatigue score when pacing goals were selected was 1.74 (SD 0.59) compared to 1.58 (SD 0.54) on days without goal selection (Mann-Whitney *U* test; *P*=.004).

Critically, the choice of which pacing strategy to use was also strongly associated with baseline symptom severity. On days when participants selected active pacing, the mean baseline fatigue score was 1.29 (SD 0.46), substantially lower than on light pacing days (mean 1.99, SD 0.50) or balanced pacing days (mean 1.78, SD 0.50). Similarly, participants selected active pacing on days when they reported a higher baseline energy score (mean 61.3, SD 18.5) compared to light pacing days (mean 47.5, SD 18.2) or balanced pacing days (mean 46.7, SD 18.2).

This pattern indicates that participants selected active pacing when feeling relatively better and light pacing when feeling worse, introducing substantial confounding by indication. While our mixed-effects models statistically controlled for these measured baseline differences, this self-selection based on symptom state complicates causal interpretation of observed associations.

### Primary Outcomes: Same-Day Effects

#### Overview

**Figure 1:**
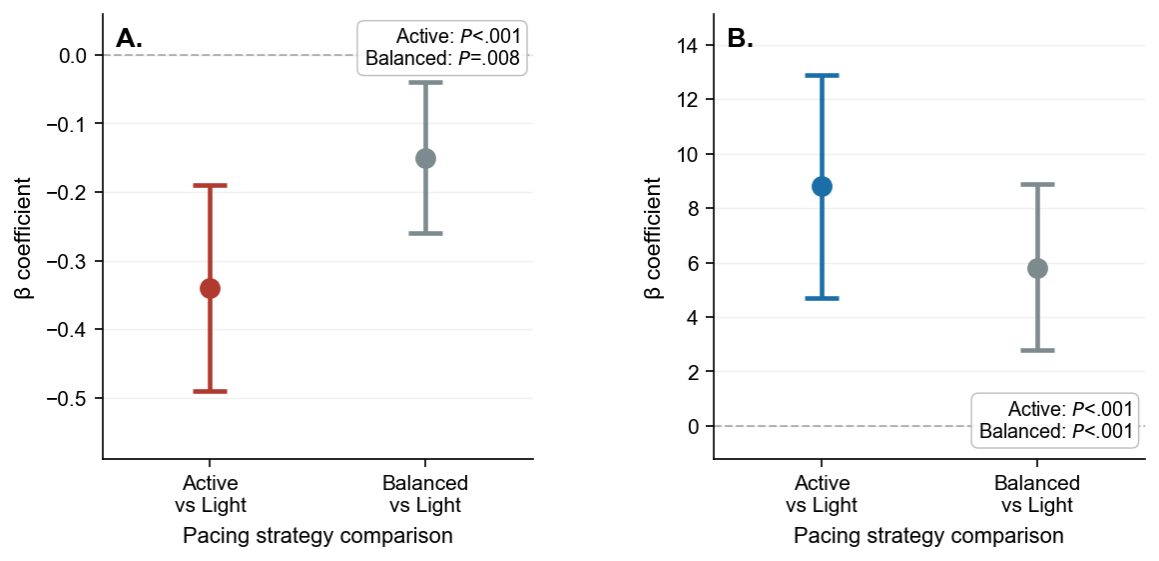
Same-day activity pacing effects on (A) fatigue and (B) energy in adults with post–COVID-19 fatigue (exploratory observational study, Newcastle, United Kingdom, 2024; n=18 participants, 584 complete-case observations): mixed-effects linear regression coefficients (β) with 95% CIs for active and balanced pacing goals compared to light pacing (reference) controlling for age and sex.

Mixed-effects linear regression results for same-day outcomes are presented in Table 2 and Figure 1.

**Table 2:** Activity pacing effects on same-day and next-day fatigue and energy outcomes in adults with post–COVID-19 fatigue: mixed-effects linear regression models (Newcastle, United Kingdom; May 2024-December 2024; n=18 participants; 584 complete-case observations)^a^.

Outcome and comparison		β coefficient (95% CI)	*P* value	Result
**Same-day fatigue**				
	Active vs light pacing	−0.34 (−0.49 to −0.19)	<.001	Significant reduction
	Balanced vs light pacing	−0.15 (−0.26 to −0.04)	.008	Significant reduction
**Same-day energy**				
	Active vs light pacing	8.8 (4.7 to 12.9)	<.001	Significant increase
	Balanced vs light pacing	5.8 (2.8 to 8.9)	<.001	Significant increase
**Next-day fatigue**				
	Active vs light pacing	−0.05 (−0.19 to 0.10)	.53	Not significant
	Balanced vs light pacing	0.04 (−0.07 to 0.15)	.47	Not significant
**Next-day energy**				
	Active vs light pacing	1.1 (−3.0 to 5.2)	.61	Not significant
	Balanced vs light pacing	−1.8 (−4.9 to 1.2)	.24	Not significant

^a^Same-day models control for age and sex only (not contemporaneous outcomes). Next-day models control for age, sex, and prior-day symptoms to account for autocorrelation. Daily step count was not included as it may represent a mediator rather than a confounder. Mixed-effects models include random intercepts for participants (n=18; intraclass correlation coefficient=0.32). Sample: 584 observations with complete data.

#### Same-Day Fatigue

Active pacing was strongly associated with reduced same-day fatigue (β=−0.34, 95% CI −0.49 to −0.19; *P*<.001) compared to light pacing after controlling for age and sex. This represents an approximately 17% reduction on the fatigue scale from 0 to 3. Balanced pacing also showed an approximately 8% significant reduction (β=−0.15, 95% CI −0.26 to −0.04; *P*=.008). Mean fatigue scores by pacing goal were 1.99 (SD 0.50) for light, 1.78 (SD 0.50) for balanced, and 1.29 (SD 0.46) for active.

#### Same-Day Energy

Both active and balanced pacing were strongly associated with increased same-day energy compared to light pacing after controlling for age and sex. Active pacing showed a β value of 8.8 (95% CI 4.7-12.9; *P*<.001), representing approximately an 18% improvement on the energy scale from 0 to 100. Balanced pacing showed a β value of 5.8 (95% CI 2.8-8.9; *P*<.001), representing approximately a 12% improvement. Mean energy scores by pacing goal were 47.5 (SD 18.2) for light, 46.7 (SD 18.2) for balanced, and 61.3 (SD 18.5) for active.

### Secondary Outcomes: Next-Day Effects

**Figure 2:**
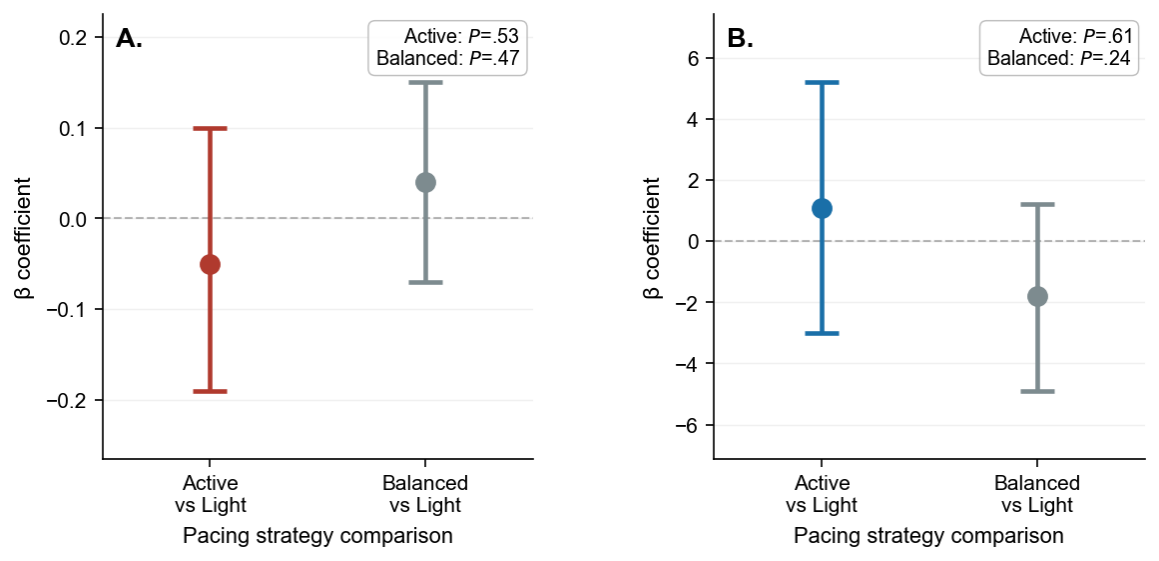
Next-day activity pacing effects on (A) fatigue and (B) energy in adults with post–COVID-19 fatigue (exploratory observational study, Newcastle, United Kingdom, 2024; n=18 participants, 584 complete-case observations): mixed-effects linear regression coefficients (β) with 95% CIs for active and balanced pacing goals compared to light pacing (reference) controlling for prior-day symptoms, age, and sex.

Next-day associations showed similar directional patterns to same-day effects but were attenuated and nonsignificant in fully adjusted models (Table 2; Figure 2). After controlling for same-day fatigue, energy, age, and sex, active pacing showed a negative but nonsignificant association with next-day fatigue (β=−0.05, 95% CI −0.19 to 0.10; *P*=.53). Mean next-day fatigue scores were 1.92 (SD 0.51) for light pacing, 1.78 (SD 0.52) for balanced pacing, and 1.30 (SD 0.46) for active pacing. Next-day energy associations were similarly small and nonsignificant (active vs light pacing: β=1.1, 95% CI −3.0 to 5.2, and *P*=.61; balanced vs light pacing: β=−1.8, 95% CI −4.9 to 1.2, and *P*=.24). These null findings suggest that the benefits of pacing strategies may be limited to same-day symptom management, although the absence of evidence of next-day benefit should not be equated with evidence of absence given the limited sample size.

### Model Diagnostics

The intraclass correlation coefficient for the next-day fatigue model was 0.32, indicating that 32% of the variance was attributable to between-person differences and 68% was attributable to within-person variation. This substantial between-person variance justified the mixed-effects modeling approach and suggested heterogeneous treatment responses.

Random intercepts varied significantly across participants (variance=0.21; *P*<.001), confirming that individuals had different baseline symptom levels even after controlling for measured covariates.

### Individual Response Heterogeneity

Of 12 participants who tried multiple pacing strategies and had sufficient data for within-person comparisons, 7 (58.3%) showed meaningful responses (≥0.3-point fatigue reduction) when using structured pacing strategies compared to their own light pacing days. A total of 41.7% (n=5) of the participants showed minimal or no benefit (<0.3-point change).

The optimal strategy varied across individuals: of the 12 participants, 4 (33.3%) benefited most from active pacing, 3 (25%) benefited most from balanced pacing, and 2 (16.7%) showed lowest fatigue with light pacing. This heterogeneity suggests that no single pacing approach is universally optimal.

### Objective Activity Verification by Pacing Strategy

To examine whether self-selected pacing goal labels corresponded to objectively different activity behaviors, we compared Fitbit-derived daily step counts and active minutes across strategy types.

Step counts differed highly significantly across pacing strategies (Kruskal-Wallis *H*=224.30; *P*<.001). Median daily steps were 3708 (IQR 2169-5678) for light pacing, 7006 (IQR 4970-8811) for balanced pacing, and 10,792 (IQR 7071-15,125) for active pacing. All pairwise comparisons were significant after Bonferroni correction (light vs balanced pacing: *P*<.001; light vs active pacing: *P*<.001; balanced vs active pacing: *P*<.001). Active minutes followed the same pattern (Kruskal-Wallis *H*=140.46; *P*<.001): median 170 (IQR 100-225), 237 (IQR 170-287), and 310 (IQR 214-406) minutes for light, balanced, and active pacing, respectively

Importantly, step counts on days when participants selected any pacing goal were not significantly different from step counts on days when no goal was selected (median 6242 vs 6304 steps; Mann-Whitney *U*=242,866; *P*=.49). This indicates that the act of selecting a pacing goal on a given day was not systematically associated with higher overall activity, partially mitigating the ascertainment bias concern that participants set goals only on their better days.

These objective activity data validate that pacing strategy labels reflected meaningfully different activity levels: active pacing days involved approximately 3 times the step count of light pacing days, consistent with the goal descriptions provided.

## Discussion

### Principal Findings

In this exploratory observational study of 19 adults with post–COVID-19 fatigue, structured activity pacing strategies (active and balanced) were associated with substantially improved same-day symptom outcomes compared to minimal pacing (light). Active pacing showed the strongest associations, with significant reductions in same-day fatigue (β=−0.34; *P*<.001) and increases in energy (β=8.8; *P*<.001) despite involving higher physical activity levels. Balanced pacing also showed significant benefits for both outcomes. Next-day effects showed weaker associations that did not reach statistical significance.

Individual heterogeneity was substantial, with 58.3% (7/12) of participants showing clinically meaningful responses to structured pacing, whereas 41.7% (5/12) showed minimal benefit. Participants demonstrated symptom-responsive behavior, selecting pacing goals more frequently on days with higher baseline fatigue (*P*=.004). This pattern of confounding by indication, combined with the observational study design, precludes causal inference about pacing effectiveness.

### Interpretation and Mechanisms

The observed associations between active pacing and improved same-day outcomes are noteworthy given that active pacing involved substantially higher physical activity. In post–COVID-19 fatigue, as in other postviral conditions, activity increases may trigger symptom exacerbation [5,21]. That active pacing was associated with reduced fatigue despite higher activity suggests that structured, goal-directed approaches may help individuals stay within their “energy envelope”—matching energy expenditure to available capacity while maintaining physical function [9].

Several mechanisms could explain these associations. First, goal setting may provide a cognitive framework for activity regulation, supporting more accurate calibration of exertion to capacity [22]. Second, proactive planning may enable activity distribution that avoids the boom-bust cycles characteristic of reactive, symptom-contingent approaches [7]. Third, successfully meeting activity goals may enhance self-efficacy, potentially modulating symptom perception [23].

However, confounding by indication complicates interpretation substantially. Participants chose active pacing when baseline fatigue was lower (mean score 1.29, SD 0.46 vs 1.99, SD 0.50 for light pacing), suggesting that they selected strategies based on symptom state. While our mixed-effects models controlled for measured baseline symptoms, unmeasured factors (eg, sleep quality, stress, and anticipated activities) could influence both pacing selection and outcomes. The observed association may partly reflect accurate symptom self-monitoring rather than a direct effect of pacing strategy.

The attenuation of effects from the same day to the next day is noted. We acknowledge that, in post–COVID-19 fatigue and related postviral conditions, delayed postexertional symptom responses can occur more than 24 hours after exertion [5]. Our study design, which examined next-day (24 hours) outcomes only, may not have captured delayed responses. This is an important limitation of this analysis that future studies should address with longer follow-up windows.

### Strengths and Limitations

Strengths include objective activity monitoring via wearable devices (reducing recall bias compared to self-reported activity questionnaires), a longitudinal within-person design capturing real-world behavior over extended periods, comprehensive statistical control for demographics and baseline symptoms using mixed-effects models, and transparent reporting of both population- and individual-level findings including nonresponders.

Critical limitations must be acknowledged. First and most importantly, the observational design with self-selected pacing precludes causal inference. Observed associations likely reflect both potential benefits of structured pacing *and* selection of pacing strategies based on baseline symptom state. Despite controlling for measured confounders, unmeasured factors (sleep quality, stress, pain levels, motivation, and anticipated social activities) could explain some or all the observed associations. Only an adequately powered randomized controlled trial can establish causality, although even then, the Hill [24] criteria for causal inference (eg, temporal sequence, dose-response, and biological plausibility) should be systematically evaluated.

Second, this study did not include a formal assessment of postexertional malaise (PEM) at enrollment. Pacing is typically indicated for individuals who experience PEM—a worsening of symptoms following physical, cognitive, or emotional exertion that may be delayed by 24 to 72 hours [5]. The proportion of participants experiencing PEM in this sample was unknown, which limits our ability to characterize the study population precisely and may affect generalizability. Future studies should formally assess PEM status using validated criteria.

Third, the pacing goal thresholds used in this study (light: <3000 steps; balanced: 3000-7000 steps; active: >7000 steps) were derived from general population activity recommendations [25] and were applied uniformly to all participants. These thresholds may not be appropriate for individuals with more severe post–COVID-19 fatigue, for whom even 3000 steps may represent substantial exertion. The mean daily step count in this sample (6821, SD 5204) suggests a relatively mild or moderately affected cohort, and findings may not generalize to individuals with more severe functional limitations. Future research should adopt individualized or adaptive pacing thresholds calibrated to each participant’s functional capacity.

Fourth, pacing goal selection occurred only on 37.4% (816/2182) of observation days, and the decision to declare a pacing goal on any given day may itself be energy dependent introducing an ascertainment bias whereby participants with sufficient energy to engage with the app were also those more likely to achieve pacing goals. Notably, objective step counts on pacing vs nonpacing days were not significantly different (median 6242 vs 6304 steps; *P*=.49), suggesting that goal selection was not simply a marker of higher-activity days. Nevertheless, unmeasured factors such as motivation, cognitive capacity, or anticipated schedule may still have influenced both goal selection and outcomes, and this potential bias cannot be fully excluded.

Fifth, the modest sample size (18 participants and 584 observation days with complete data) limited statistical power, particularly for subgroup analyses. The responder analysis included only 66.7% (12/18) of the participants, who tried multiple strategies, as 33.3% (6/18) of the participants limited themselves to 1 or 2 approaches. While this reflects real-world adherence patterns, it limits within-person conclusions about optimal strategies.

Sixth, we used a study-specific 4-point ordinal fatigue scale and a visual analog scale for energy rather than validated instruments such as the Fatigue Severity Scale or Chalder Fatigue Scale. While these brief scales were chosen to minimize participant burden and maximize daily adherence, their psychometric properties in this context are unknown. Future studies should incorporate validated fatigue and quality of life instruments alongside brief daily measures.

Seventh, we treated ordinal fatigue ratings as continuous, an approximation that may not fully capture ordinal structure. Mixed-effects ordinal logistic models would be more appropriate but require substantially larger samples for stable estimation [18].

Finally, given the exploratory nature of this study, we did not adjust for multiple comparisons [26]. Therefore, the findings should be interpreted as hypothesis-generating associations requiring confirmation in preregistered confirmatory studies rather than as definitive evidence.

### Comparison With Previous Literature

Our findings align with emerging evidence on activity pacing in postviral fatigue. Patient-reported research in ME/CFS has distinguished symptom-contingent (reactive) from preplanned (proactive) pacing, with some literature suggesting potential benefits of more structured, preplanned approaches [7,9,10]. Our active pacing approach aligns conceptually with preplanned strategies and was associated with better same-day outcomes in this study. However, our population comprised individuals with post–COVID-19 fatigue rather than ME/CFS, and direct comparisons should be made cautiously given the differences between these conditions.

The observed pattern of higher activity alongside lower fatigue on active pacing days is consistent with the “energy envelope” framework, which proposes that aligning activity with available energy may support symptom management [9]. Structured goal setting may help facilitate this balance, although underlying mechanisms remain unclear.

Post–COVID-19 fatigue may differ from ME/CFS in important ways, including evidence of variable symptom trajectories and potential for recovery in some individuals [12,13]. Whether pacing strategies developed in ME/CFS populations translate to post–COVID-19 fatigue populations requires further empirical investigation.

### Clinical and Research Implications

These exploratory findings suggest that active, goal-directed pacing may warrant discussion as part of post–COVID-19 fatigue self-management, particularly for same-day symptom control. Rather than universally advising activity minimization, clinicians might discuss setting sustainable activity goals aligned with patients’ individual energy levels. Digital tools integrating activity monitoring and symptom tracking could facilitate such approaches, although efficacy remains to be established.

Randomized controlled trials are needed to establish causality. Future research should (1) randomize participants to different pacing strategies with adequate sample sizes and formal PEM assessment at enrollment; (2) blind outcome assessors where feasible; (3) use validated fatigue outcome measures; (4) examine mechanisms through detailed assessment of activity patterns, sleep, and psychological factors; (5) identify baseline characteristics predicting who benefits from which strategy; (6) use individualized activity thresholds rather than population-level targets; and (7) test across illness severity levels and diverse populations to establish generalizability.

Long-term studies could assess whether sustained pacing strategies are associated with functional recovery beyond symptom management (eg, return to work and quality of life). Mechanistic studies using objective markers of immune, metabolic, or autonomic function could elucidate biological pathways, although such studies would need to carefully establish temporal relationships given confounding challenges in observational designs.

### Conclusions

In this exploratory observational study using wearable devices and mobile symptom tracking, structured activity pacing strategies (active and balanced) were associated with improved same-day fatigue and energy in individuals with post–COVID-19 fatigue despite involving higher physical activity levels. Next-day effects were attenuated and nonsignificant. However, substantial confounding by indication, wherein participants selected active pacing when feeling relatively better, combined with individual heterogeneity (approximately half, 7/12, 58.3% of the participants showed meaningful responses), the absence of PEM assessment, and the use of nonvalidated symptom scales limit causal interpretation. These preliminary associations suggest that structured, goal-directed pacing warrants rigorous testing in adequately powered randomized controlled trials with formal PEM assessment, validated outcome measures, and individualized activity thresholds to establish efficacy and optimize implementation for post–COVID-19 fatigue care.

## Appendix

Multimedia Appendix 1 STROBE checklist.

## Abbreviations

ME/CFS: myalgic encephalomyelitis/chronic fatigue syndrome
PEM: postexertional malaise
STROBE: Strengthening the Reporting of Observational Studies in Epidemiology

## Data Availability

The datasets generated or analyzed during this study are available from the corresponding author on reasonable request**.**
