# Supplementary material for: Wearable- and Mobile App–Based Activity Pacing and Fatigue Management in Post–COVID-19 Condition: Exploratory Observational Study
Source: JMIR Form Res. 2026 May 29;10:e91829. doi: 10.2196/91829 (PMC13263657; doi:10.2196/91829)
Supplement: Multimedia Appendix 1 [file formative_v10i1e91829_app1.pdf]

STROBE Statement—checklist of items that should be included in reports of observational studies

Manuscript title: Wearable and Mobile App-Based Activity Pacing and Fatigue Management in Post-COVID-19 Condition: An Exploratory Observational Study

Manuscript number: 91829 **|** JMIR Formative Research

Study design: Exploratory observational cohort study (repeated measures)

Item No	Recommendation	Page No
	**Title and abstract**	
**1**	(a) Indicate the study's design with a commonly used term in the title or the abstract	Title ("Exploratory Observational Study"); Abstract, Methods paragraph
	(b) Provide in the abstract an informative and balanced summary of what was done and what was found	Abstract — Background, Objective, Methods, Results, Conclusions
	**Introduction**	
	Background/rationale	
**2**	Explain the scientific background and rationale for the investigation being reported	Introduction, paragraphs 1–4
	Objectives	
**3**	State specific objectives, including any prespecified hypotheses	Introduction, final paragraph: hypothesis stated explicitly
	**Methods**	
	Study design	
**4**	Present key elements of study design early in the paper	Methods, "Study Design and Setting", first sentence
	Setting	
**5**	Describe the setting, locations, and relevant dates, including periods of recruitment, exposure, follow-up, and data collection	Methods, "Study Design and Setting" and "Participants and Recruitment": Newcastle University, UK; May–December 2024
	Participants	
**6**	(a) Cohort study—Give the eligibility criteria, and the sources and methods of selection of participants. Describe methods of follow-up	Methods, "Participants and Recruitment": 3 eligibility criteria listed; 2 recruitment channels described; remote follow-up via app over observation period
	(b) Cohort study—For matched studies, give matching criteria and number of exposed and unexposed	N/A — not a matched study
	Variables	
**7**	Clearly define all outcomes, exposures, predictors, potential confounders, and effect modifiers. Give	Methods, "Outcomes" and "Data Analysis": primary/secondary outcomes defined; pacing

	diagnostic criteria, if applicable	goal as exposure; age and sex as covariates; prior-day symptoms as confounders in next-day models
	Data sources/measurement	
**8***	For each variable of interest, give sources of data and details of methods of assessment (measurement). Describe comparability of assessment methods if there is more than one group	Methods, "Data Collection": Fitbit Inspire 3 for steps/active minutes; FatigueSense app for fatigue (0–3 ordinal scale) and energy (0–100 VAS); pacing goal selection described with goal descriptions verbatim
	Bias	
**9**	Describe any efforts to address potential sources of bias	Methods, "Confounding-by-Indication Assessment"; mixed-effects models with random intercepts used throughout; Results, "Objective Activity Verification" (step count validation); Discussion, "Strengths and Limitations"
	Study size	
**10**	Explain how the study size was arrived at	No formal a priori sample size calculation — convenience sample from PPIE cohort and online communities. Acknowledged as a limitation in Discussion, "Strengths and Limitations"
	Quantitative variables	
**11**	Explain how quantitative variables were handled in the analyses. If applicable, describe which groupings were chosen and why	Methods, "Data Analysis": fatigue treated as continuous (ordinal approximation acknowledged as limitation); energy as continuous (0–100); age as 4 categorical groups; pacing goal as categorical (3 levels, Light as reference)
	Statistical methods	
**12**	(a) Describe all statistical methods, including those used to control for confounding	Methods, "Mixed-Effects Linear Models": mixed-effects linear regression with random intercepts; covariates specified separately for same-day and next-day models; Mann-Whitney U for confounding-by-indication; Kruskal-Wallis and pairwise Mann-Whitney for step count validation
	(b) Describe any methods used to examine subgroups and interactions	Methods, "Responder Analysis": within-person responder analysis defined (≥0.3-point fatigue reduction threshold; participants trying multiple strategies)
	(c) Explain how missing data were addressed	Methods, "Data Inclusion Criteria": complete-case analysis; participants with <3 observation days excluded; 584 complete-case observations from 18 participants
	(d) Cohort study—If applicable, explain how loss to follow-up was addressed	Results, "Participants": all 19 enrolled participants contributed data; 1 excluded for insufficient observations (1 day); observation days per participant reported (mean 115, range 1–146)
	(e) Describe any sensitivity analyses	No formal sensitivity analyses conducted — exploratory study. Acknowledged as a limitation in Discussion, "Strengths and Limitations"

	Results	
	Participants	
**13***	(a) Report numbers of individuals at each stage of study—eg numbers potentially eligible, examined for eligibility, confirmed eligible, included in the study, completing follow-up, and analysed	Results, "Participants": 19 enrolled; 1 excluded; 18 in complete-case mixed-effects analyses; 584 observations with complete data
	(b) Give reasons for non-participation at each stage	Results, "Participants": 1 participant excluded (contributed only 1 observation day — insufficient for within-person estimation). Reasons for non-response at recruitment not available (community-based; acknowledged)
	(c) Consider use of a flow diagram	No flow diagram included; participant numbers described in text. A flow diagram can be added if required by the editor.
	Descriptive data	
**14***	(a) Give characteristics of study participants (eg demographic, clinical, social) and information on exposures and potential confounders	Results, Table 1: age, sex, observation days, pacing goal usage by strategy, symptom scores (fatigue and energy), Fitbit step count and active minutes
	(b) Indicate number of participants with missing data for each variable of interest	Methods, "Data Inclusion Criteria": 816 total pacing days; 584 included after excluding missing covariate data (232 excluded). Step count data missing for 764 of 2,182 observation days (noted in Results, "Objective Activity Verification")
	(c) Cohort study—Summarise follow-up time (eg, average and total amount)	Results, "Participants" and Table 1: mean 115 days per participant (range 1–146); total 2,182 observation days
	Outcome data	
**15***	Cohort study—Report numbers of outcome events or summary measures over time	Results: mean fatigue and energy by pacing strategy reported in text; Table 2 reports regression coefficients with 95% CIs for all outcomes;
	Main results	
**16**	(a) Give unadjusted estimates and, if applicable, confounder-adjusted estimates and their precision (eg, 95% confidence interval). Make clear which confounders were adjusted for and why they were included	Results: unadjusted descriptive means by strategy reported in text ("Mean fatigue levels by pacing goal were..."); adjusted estimates in Table 2 with 95% CIs; covariates and rationale stated in Methods, "Model specification"
	(b) Report category boundaries when continuous variables were categorized	Methods, "Symptom Reporting and Pacing Goal Selection": step count targets per goal (Light <3,000; Balanced 3,000–7,000; Active >7,000); age group boundaries stated in Table 1
	(c) If relevant, consider translating estimates of relative risk into absolute risk for a meaningful time period	Results: regression coefficients reported on original scale (fatigue 0–3; energy 0–100); percentage change interpretations provided in text (e.g., "approximately 17% reduction on the 0–3 fatigue scale")
	Other analyses	
**17**	Report other analyses done—eg analyses of subgroups and interactions, and sensitivity analyses	Results, "Individual Response Heterogeneity": within-person responder analysis; Results, "Objective Activity Verification by Pacing

		Strategy": step count comparison across strategies and pacing vs non-pacing days (Kruskal-Wallis, pairwise Mann-Whitney)
	**Discussion**	
	Key results	
**18**	Summarise key results with reference to study objectives	Discussion, "Principal Findings": key results summarised with reference to original hypothesis
	Limitations	
**19**	Discuss limitations of the study, taking into account sources of potential bias or imprecision. Discuss both direction and magnitude of any potential bias	Discussion, "Strengths and Limitations": 7 limitations discussed including confounding-by-indication (direction: likely upward bias for Active pacing), missing PEM assessment, one-size-fits-all thresholds, ascertainment bias, sample size, non-validated scales, ordinal-as-continuous approximation
	Interpretation	
**20**	Give a cautious overall interpretation of results considering objectives, limitations, multiplicity of analyses, results from similar studies, and other relevant evidence	Discussion, "Interpretation and Mechanisms", "Comparison with Previous Literature"; Conclusions: findings framed as associations not causal effects; multiple limitations foregrounded; no multiple comparison adjustment applied and acknowledged
	Generalisability	
**21**	Discuss the generalisability (external validity) of the study results	Discussion, "Strengths and Limitations" (mild cohort caveat; step count mean 6,821 suggesting moderate impairment) and "Clinical and Research Implications": generalisability to severe post-COVID fatigue and non-UK populations discussed
	**Other information**	
	Funding	
**22**	Give the source of funding and the role of the funders for the present study and, if applicable, for the original study on which the present article is based	Funding Statement (separate section): NIHR Newcastle BRC, EPSRC (EP/X031012/1; EP/X036146/1), IMI2 IDEA-FAST (853981), LifeArc, MNDA, MyName5Doddie Foundation. Funders had no role in study design, data collection, analysis, interpretation, or decision to publish.

**Give information separately for cases and controls in case-control studies and, if applicable, for exposed and unexposed groups in cohort and cross-sectional studies.*

Note: An Explanation and Elaboration article discusses each checklist item and gives methodological background and published examples of transparent reporting. The STROBE checklist is best used in conjunction with this article (freely available on the Web sites of PLoS Medicine at http://www.plosmedicine.org/, Annals of Internal Medicine at http://www.annals.org/, and Epidemiology at http://www.epidem.com/). Information on the STROBE Initiative is available at www.strobe-statement.org.
